# Intraoperative Discovery of Placenta Percreta With Bladder Invasion During Emergency Cesarean Section Following Cardiac Arrest: A Case Report

**DOI:** 10.7759/cureus.111291

**Published:** 2026-06-22

**Authors:** Bouchra Chahboun, Amine Achraf Majit, Ikram Mekkaoui, Ghizlane El Aidouni, Ahmed Mimouni, Houssam Bkiyar

**Affiliations:** 1 Intensive Care Unit, Faculty of Medicine and Pharmacy of Oujda, Mohammed First University, Oujda, MAR; 2 Intensive Care Unit, Mohammed VI University Hospital, Oujda, MAR; 3 Laboratory of Anatomy, Microsurgery and Surgery Experimental and Medical Simulation (LAMCESM), Faculty of Medicine and Pharmacy of Oujda, Mohammed First University, Oujda, MAR; 4 Physical Medicine and Rehabilitation, Faculty of Medicine and Pharmacy, Mohammed First University, Oujda, MAR; 5 Physical Medicine and Rehabilitation, Faculty of Medicine and Pharmacy, Mohammed VI University Hospital, Oujda, MAR; 6 Gynecology, Mohammed VI University Hospital, Oujda, MAR

**Keywords:** cesarean hysterectomy, critical care obstetrics, obstetric hemorrhage, placenta accreta spectrum (pas), surgical case reports

## Abstract

Placenta percreta, the most severe form of placenta accreta spectrum (PAS), is a rare but life-threatening obstetric condition in which abnormal placental invasion extends beyond the uterine myometrium into adjacent organs, most frequently the urinary bladder. Preoperative imaging may suggest the diagnosis yet substantially underestimate the true depth of invasion, a discrepancy that carries potentially catastrophic consequences. We report the case of a 34-year-old woman with two prior cesarean sections who presented at 25 weeks of gestation in active preterm labor with a dichorionic-diamniotic twin pregnancy. Antenatal ultrasound raised suspicion for PAS, but full-thickness bladder wall invasion was confirmed only upon surgical exploration. After delivery of both twins, a total hysterectomy with en bloc partial posterior cystectomy and bilateral ureteral stenting was required. The procedure was complicated by massive hemorrhage, refractory hemodynamic shock, and an asystolic cardiac arrest from which return of spontaneous circulation was achieved within approximately one minute following immediate cardiopulmonary resuscitation and intravenous epinephrine as part of a coordinated multidisciplinary resuscitation effort. The patient achieved complete neurological recovery and was discharged on postoperative day 10. This case underscores that any sonographic suspicion of PAS, regardless of apparent severity, mandates maximal multidisciplinary preparedness and that survival with full neurological recovery following intraoperative cardiac arrest is attainable when resuscitation is prompt and protocol-driven.

## Introduction

Placenta accreta spectrum (PAS) encompasses a range of abnormal placental attachment disorders. In placenta accreta, the chorionic villi are abnormally adherent to the myometrium without invasion. Placenta increta involves penetration into the myometrial layer, whereas placenta percreta represents the most advanced form, extending through the uterine wall and occasionally involving adjacent pelvic organs, most commonly the bladder [1,2]. Percreta is the rarest and most morbid PAS variant, carrying the highest rates of hemorrhage-related maternal morbidity and mortality [3].

PAS incidence has risen markedly, driven by increasing cesarean delivery rates [4,5]. Prior uterine surgery combined with placenta previa constitutes the dominant risk-factor constellation [5-7]. When diagnosed antenatally, planned cesarean hysterectomy at 34-36 weeks at a tertiary center is the standard of care [8,9].

Despite overall ultrasound sensitivity of approximately 91% for PAS detection [10,11], true invasion depth is frequently underestimated, particularly in emergency settings or multiple gestations [3,10]. We present a case of intraoperatively confirmed placenta percreta with bladder invasion during emergency cesarean delivery for a twin pregnancy at 25 weeks, complicated by massive hemorrhage and cardiac arrest, with successful maternal survival.

## Case presentation

Presentation and workup

**Figure 1:**
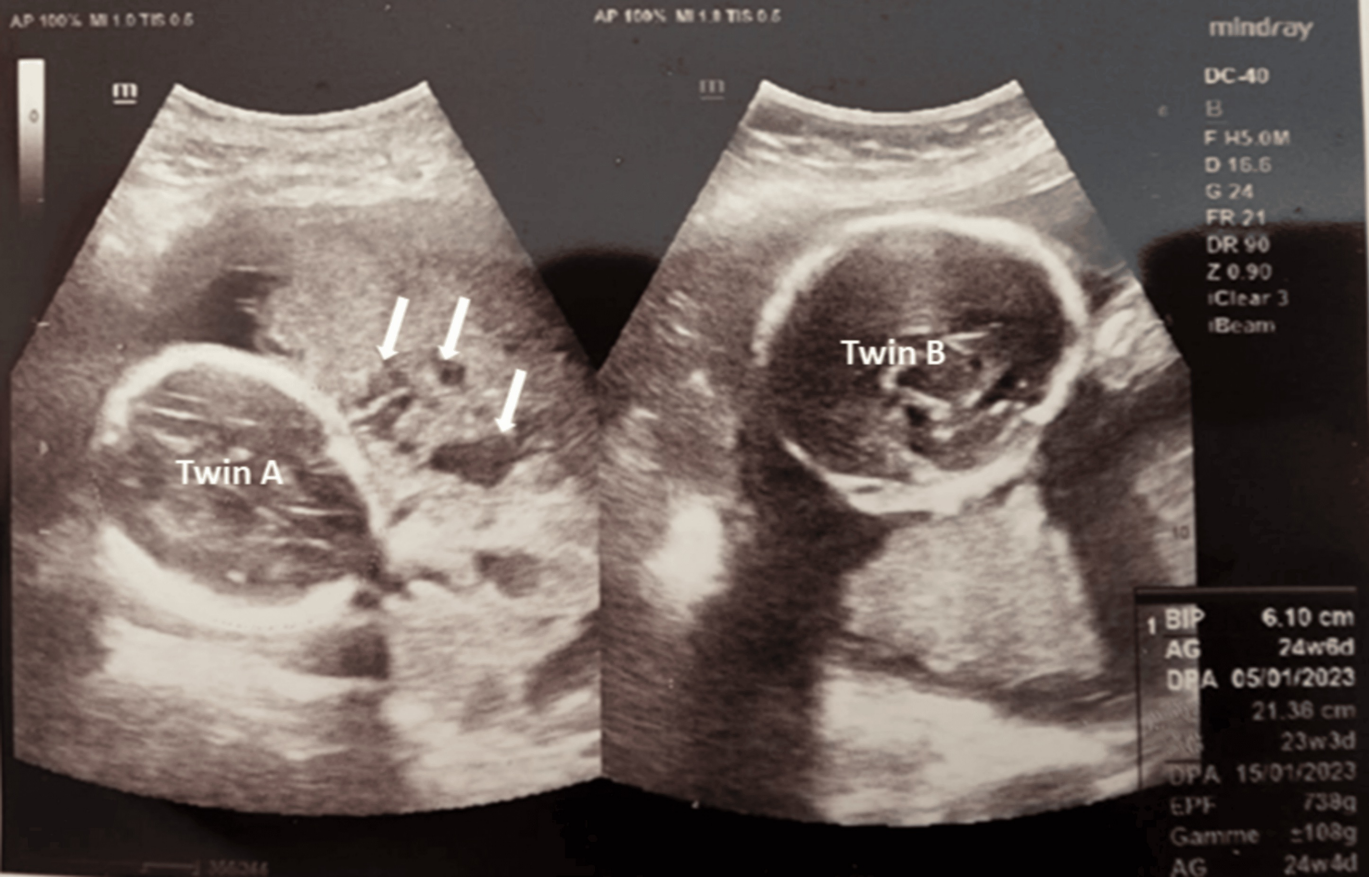
Preoperative Obstetric Ultrasound Findings in Twin Pregnancy With Suspected Placenta Accreta Spectrum Preoperative obstetric ultrasound at 24 weeks + 6 days. Left panel (Twin A): anterior placenta previa with irregular intraplacental lacunae (arrows) suggestive of placenta accreta spectrum (PAS). Reliable assessment of invasion depth was limited by the emergency setting and complexity of the twin gestation. Right panel (Twin B): normal fetal cranial biometry. BPD: biparietal diameter; GA: gestational age; EFW: estimated fetal weight; PAS: placenta accreta spectrum.

A 34-year-old woman (G3P2) with two prior low-transverse cesarean sections performed for fetal macrosomia presented at 25 weeks of gestation in active preterm labor with a bichorionic-biamniotic (BCBA) twin pregnancy. An external ultrasound performed 3 weeks prior had shown no features suggestive of PAS. On admission, she was hemodynamically stable (HR 90 bpm, BP 118/79 mmHg, SpO₂ 98%). Baseline investigations revealed a mildly reduced hemoglobin level, a platelet count within normal limits, and a prothrombin time within the normal range (Table 1). Repeat ultrasound identified anterior placenta previa with multiple irregular intraplacental lacunae consistent with PAS (Figure 1); reliable assessment of invasion depth was precluded by the twin gestation and the emergency context. Preoperative MRI and transfer to a tertiary center were not feasible given the acuity of presentation. Upon identification of suspected PAS, immediate multidisciplinary preparation was initiated: the obstetric, urological, and anesthesiology teams were placed on standby; cross-matched packed red blood cells, fresh frozen plasma, and platelets were preordered; a massive transfusion protocol was made available; and contingency planning for hemorrhagic shock and emergency hysterectomy was explicitly discussed with all operative team members. Emergency cesarean section was undertaken under spinal anesthesia, with the full surgical team on alert for potential PAS complications.

**Table 1:** Admission and Postoperative Laboratory Values POD: postoperative day; PT: prothrombin time; INR: international normalized ratio; PaCO₂: arterial CO₂ partial pressure; PaO₂: arterial O₂ partial pressure; HCO₃⁻: bicarbonate. Dashes indicate values not collected at that time point.

Parameter	Admission	Postoperative (POD 1)	Reference Range
Hemoglobin (g/dL)	8.6	8.8	12.0-16.0
Hematocrit (%)	—	25.1	36-48
Platelets (×10³/µL)	203	103	150-400
PT (%) / INR	100 / 0.97	100 / —	70-120 / 0.8-1.2
Fibrinogen (g/L)	—	2.8	2.0-4.5
Lactate (mmol/L)	—	1.72	<2.0
pH	—	7.42	7.35-7.45
PaCO₂ (mmHg)	—	39.4	35-45
PaO₂ (mmHg)	—	273	80-100
HCO₃⁻ (mmol/L)	—	25.4	22-26
Serum creatinine (mg/dL)	0.5	—	0.5-1.1

Intraoperative course

Following large-bore intravenous access and crystalloid preload, a subarachnoid block was performed at the L3-L4 interspace using hyperbaric bupivacaine 0.5% (10 mg) and fentanyl (25 µg). Intraoperative findings revealed placenta percreta with active hemorrhage, protrusion of the gestational sac through the right anterolateral uterine wall, and frank full-thickness bladder wall invasion that markedly exceeded preoperative expectations. Both twins were delivered promptly (Twin 1: Apgar 8/9; Twin 2: Apgar 8/10) and transferred to the neonatal intensive care unit (NICU).

General anesthesia was immediately induced using etomidate (0.3 mg/kg), rocuronium (1.2 mg/kg), and fentanyl (2 µg/kg), followed by orotracheal intubation. Ultrasound-guided right internal jugular catheterization and left radial arterial line placement were established. Given dense placental adherence to the posterior bladder wall that precluded safe separation, total hysterectomy with en bloc partial posterior cystectomy was performed. The urology team completed bladder reconstruction with bilateral ureteral stenting via transvesicoparietal routes, and abdominal packing was placed.

Hemorrhage, cardiac arrest, and resuscitation

Progressive hemodynamic deterioration ensued. Immediately preceding the cardiac arrest, the clinical picture was characterized by sustained systolic blood pressure below 90 mmHg despite vasopressor initiation, an estimated blood loss of 3,500 mL, and progressive bradycardia representing the terminal hemodynamic trajectory. Norepinephrine was initiated at 5 µg/min. A balanced 1:1:1 massive transfusion protocol (MTP) was activated, comprising seven units each of packed red blood cells, fresh frozen plasma, and platelets, with deliberate crystalloid restriction to prevent dilutional coagulopathy. Despite these measures, progressive bradycardia evolved into asystolic cardiac arrest. It should be noted that intra-arrest arterial blood gas data were not available; post-resuscitation laboratory values (Table 1, POD 1) reflect the post-MTP resuscitation state and should not be interpreted as peri-arrest values. Immediate cardiopulmonary resuscitation (CPR) with chest compressions and intravenous epinephrine (1 mg) achieved return of spontaneous circulation (ROSC) within approximately one minute. Vasopressor support was discontinued within 30 minutes, with no further requirement thereafter. Total operative time was 3 hours and 30 minutes.

Postoperative course

**Figure 2:**
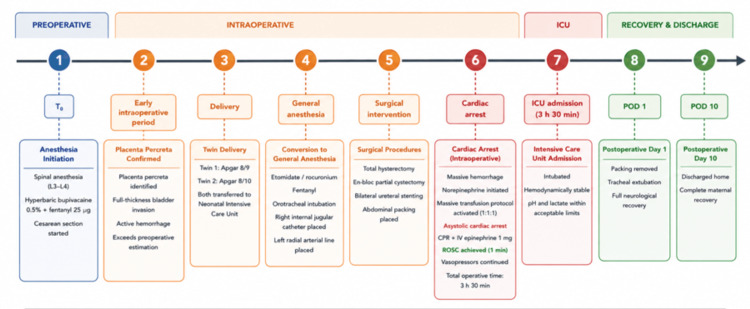
Clinical Timeline of Major Perioperative Events During Emergency Cesarean Section for Placenta Percreta With Bladder Invasion This clinical timeline illustrates the sequence of major perioperative events during emergency cesarean section for placenta percreta with bladder invasion. The timeline summarizes anesthetic management, intraoperative findings, twin delivery, conversion to general anesthesia, definitive surgical treatment, intraoperative cardiac arrest and resuscitation, intensive care unit admission, postoperative recovery, and hospital discharge. CPR: cardiopulmonary resuscitation; EBL: estimated blood loss; FFP: fresh frozen plasma; ICU: intensive care unit; MTP: massive transfusion protocol; NICU: neonatal intensive care unit; PLT: platelets; POD: postoperative day; PRBCs: packed red blood cells; ROSC: return of spontaneous circulation. Image created using MS PowerPoint (Microsoft Corporation, Redmond, Washington).

The patient was transferred to the intensive care unit (ICU) intubated and hemodynamically stable. Arterial blood gas analysis confirmed adequate metabolic resuscitation, with pH and lactate levels within acceptable postoperative limits (Table 1). Full neurological recovery was documented within 24 hours based on serial bedside neurological evaluations performed by the attending intensivist, including assessment of the Glasgow Coma Scale (GCS), motor responses, and cranial nerve function. No formal neuroimaging was obtained given the clinically complete and rapid neurological recovery, consistent with a brief, promptly resuscitated cardiac arrest in a young patient. Abdominal packing was removed, and tracheal extubation was performed on postoperative day (POD) 1. The subsequent postoperative course was uncomplicated, and the patient was discharged home on POD 10. The chronological sequence of perioperative events across the preoperative, intraoperative, postoperative ICU, and recovery phases is summarized in Figure 2. 

Patient perspective

The patient described the ICU admission as emotionally demanding. She expressed progressive recovery after discharge and relief regarding the favorable status of her twins, whom she was unable to visit during the initial postoperative days.

Ethics and consent

Written informed consent for publication was obtained in accordance with the Declaration of Helsinki. Institutional ethical approval was not required for a de-identified single case report per applicable national regulations.

## Discussion

Diagnostic limitations

Intraplacental lacunae, as identified in this case, are recognized sonographic markers of PAS but do not reliably predict percreta-level invasion or bladder penetration [10]. MRI provides superior tissue characterization [12] but was unavailable in this emergency context. The discrepancy between the normal external ultrasound performed three weeks prior to admission and the intraoperative findings of frank percreta-level invasion warrants cautious interpretation. While dynamic progression of placental invasion over time remains a plausible explanation, several alternative factors must also be considered: interobserver variability and operator-dependent limitations in ultrasound interpretation; the technical challenges of reliably assessing invasion depth in the context of twin gestation, which can obscure anatomical landmarks; and the possibility that the initial examination was performed without dedicated PAS-protocol imaging. No single explanation is sufficient, and the observed discrepancy likely reflects a combination of these factors. Any sonographic suspicion of PAS must therefore trigger maximal multidisciplinary activation, as intraoperative findings may far exceed preoperative imaging predictions [8,9].

Surgical strategy

Dense bladder adherence precluded conservative tissue separation, making en bloc hysterectomy with partial cystectomy the only safe surgical option, consistent with published percreta experience [13]. Conservative in situ placental retention carries high rates of secondary hemorrhage and organ injury [14,15] and is contraindicated in the setting of active hemorrhage. Prophylactic endovascular adjuncts [16] were unavailable in this unplanned emergency setting, as the acuity of the clinical presentation precluded the preoperative planning and vascular access required for such interventions. This represents a recognized limitation of emergency PAS management and reinforces the importance of timely antenatal diagnosis enabling planned tertiary-level care. Intraoperative urological involvement for bladder reconstruction and ureteral protection was essential to achieving a safe outcome [9].

Anesthesia and transfusion

Neuraxial anesthesia was appropriate for the initial hemodynamically stable phase [8,17]; urgent conversion to general anesthesia upon hemorrhage escalation is well-established practice in this context [17]. The balanced 1:1:1 transfusion strategy, supported by mechanistic evidence from the PROPPR randomized clinical trial conducted in trauma patients [18], has been widely adopted in obstetric massive hemorrhage management and is endorsed by national obstetric hemorrhage consensus bundles [19]. While direct extrapolation from a trauma population to obstetric hemorrhage requires caution, given differences in patient physiology, coagulopathy patterns, and hemorrhage kinetics, the shared hemostatic rationale of preventing dilutional coagulopathy through early balanced blood product administration supports its application in PAS-related hemorrhage in the absence of dedicated obstetric RCT data. Deliberate crystalloid restriction throughout was key to preventing dilutional coagulopathy.

Cardiac arrest

The asystolic arrest in this case most plausibly reflects a hemorrhagic-hypoxic mechanism with progressive preload failure, supported by the documented hemodynamic trajectory: sustained hypotension refractory to vasopressors, 3,500 mL estimated blood loss, and progressive bradycardia immediately preceding asystole. However, it must be acknowledged that the exact mechanism of arrest remains speculative in the absence of continuous intraoperative monitoring records and intra-arrest laboratory data; other contributory mechanisms, including vagal responses, anesthetic effects, or electrolyte disturbances, cannot be definitively excluded. Rapid ROSC within approximately one minute, early vasopressor cessation, and complete neurological recovery within 24 hours are consistent with a brief, effectively resuscitated circulatory event. This outcome reinforces the imperative for preprocedural cardiac arrest readiness in all PAS procedures, including pre-drawn epinephrine, immediate defibrillator availability, and clearly designated CPR roles [9,19].

Limitations

Limitations of this report include its retrospective single-case design, the absence of preoperative MRI, and the use of estimated rather than measured blood loss. Histopathological confirmation of the depth of placental invasion is not reported. Neurological recovery was assessed by serial bedside clinical evaluation; no formal neuropsychological testing or neuroimaging was performed, and the absence of standardized neurological scoring represents a limitation. Intra-arrest laboratory data were unavailable, precluding definitive characterization of the mechanism of cardiac arrest. Accordingly, the findings and outcomes may not be generalizable across all clinical settings or resource environments.

## Conclusions

Placenta percreta with bladder invasion may substantially exceed preoperative sonographic estimation. Any PAS suspicion, irrespective of grade, demands immediate multidisciplinary preparedness. In unplanned emergencies, simultaneous obstetric, urological, and critical care capacity is life-saving. Full maternal neurological recovery following intraoperative asystolic cardiac arrest is achievable when CPR is initiated without delay and resuscitation adheres to evidence-based protocols.
